# Protocol for an *in vivo* CRISPR screen for germinal center B cells in mice using ecotropic retrovirus

**DOI:** 10.1016/j.xpro.2026.104586

**Published:** 2026-05-22

**Authors:** Jun P. Hong, Michel C. Nussenzweig

**Affiliations:** 1 Laboratory of Molecular Immunology, The Rockefeller University, New York, NY 10065, USA; 2 Howard Hughes Medical Institute (HHMI), The Rockefeller University, New York, NY 10065, USA

**Keywords:** CRISPR, High Throughput Screening, Immunology, Model Organisms, Molecular Biology

## Abstract

The lack of an *in vitro* model that recapitulates germinal center (GC) B cell biology necessitates the use of animal models for genetic studies. Here, we present a protocol for an *in vivo* CRISPR screen for GC B cells in mice using an ecotropic retrovirus. We describe steps for constructing a single guide (sgRNA) library and performing a genetic screen in a mouse model of protein immunization, including procedures for sequencing and data analysis.

## Highlights

• Guidance on CRISPR-Cas9 genetic screens in *de facto* mouse germinal center (GC) B cells
• Instructions for cloning and producing an ecotropic retrovirus sgRNA library
• Immunization to generate GCs composed of library-containing antigen-specific B cells
• Procedures for genomic DNA extraction for sequencing and analysis

## Abstract (editor highlights)

Publisher’s note: Undertaking any experimental protocol requires adherence to local institutional guidelines for laboratory safety and ethics.

## Graphical abstract

**Figure:**
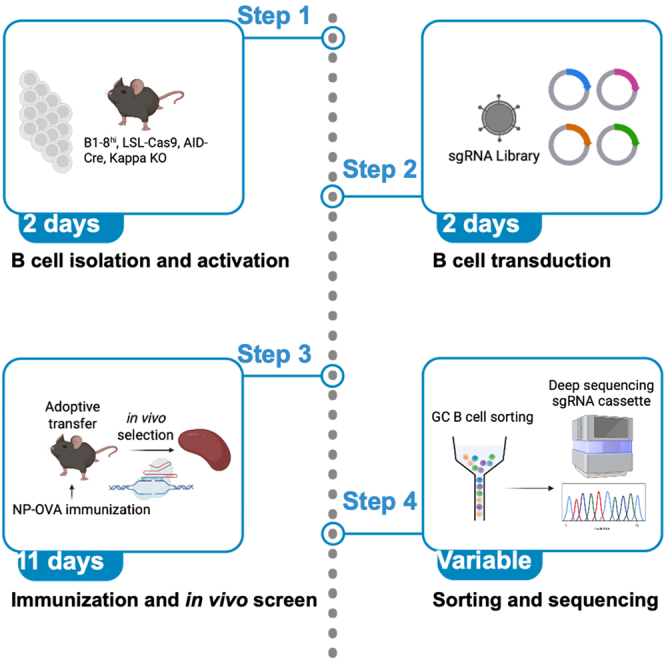

## Before you begin

This protocol is optimized for *in vivo* CRISPR screening in mouse GC B cells.

### Innovation

This protocol utilizes the optimized existing CRISPR screen tools, which include the modified sgRNA scaffold sequence,^1^ sequencing/index primer,^2^ and the analysis tools.^3^ The advancement is that this protocol merges these tools and optimize them for mouse GC B cells.

### Institutional permissions

All animal procedures were approved by the the Rockefeller University Institutional Animal Care and Use Committee, protocol number 24037-H. Users of this protocol should note that permissions for experiments on live vertebrates must be acquired in advance from the relevant institutions. This protocol requires the use of replication-incompetent lentivirus. All experiments involving potentially pathological agents must be approved by an Institutional Biosafety Committee. Local and governmental regulations concerning handling of (genetically modified) infectious agents must also be obeyed and the respective procedures authorized and carried out under the appropriate biosafety conditions.

### Pooled single guide RNA library cloning

**Timing: 3 days**

When designing sgRNA library, the user is advised to consider the size of experiment and the number of sgRNA per gene. The experiment size will depend on various factors including the number of genes to be tested, the transduction efficiency, engraftment, and GC recruitment. Typically, the larger the number of sgRNAs for each gene, the more reliable discovery rates will be.^4^^,^^5^ 5—10 sgRNAs may be chosen for each gene without significantly compromising the assay performance.^4^ Eight perturbations per gene is suggested as this number will allow capturing of minor effects while still maintaining feasible library size depending on the number genes being tested.^4^ Optimized mouse-targeting sgRNA library is available in Wang et. al.^6^ Include at least 50 negative control sgRNAs targeting intergenic regions. The design principles discussed in this protocol are specific to the retroviral system. This protocol is intended for a library size of up to 1,400 sgRNAs.

1. Clone pooled sgRNA library following the manufacture’s instruction for NEBuilder HiFi DNA Assembly and NEB Stable Competent E. coli (High Efficiency).
  a. Use 0.005 pmol of BbsI-digested pMP71-sgEmpty vector and 5 pmol DNA oligo pool (70 bp; IDT oPools).
  b. Incubate in a total volume of 20 μL for 1 h at 50 °C.

***Note:*** Each oligo contains 20 bp targeting sequence of sgRNA (or 21 bp after prepending G if the sequence does not already start with G) flanked by 5′ overhang sequence (tatcttgtggaaaggacgaaacacc) and 3′ overhang sequence (gtttaagagctatgctggaaacagc).

***Note:*** pMP-71-sgEmpty vector utilizes optimized scaffold to improve the efficiency of gene editing.^1^

***Note:*** During the initial stage of protocol development, the authors compared high-efficiency chemical transformation (#C3040H) with electroporation (Endura Competent Cells; #60242-1) and did not observe significant differences between the two systems. The authors did not further optimize the electroporation conditions but opted for high-efficiency chemical transformation given that this protocol is intended for library size up to 1,400 sgRNAs. However, in case the users observe incomplete representation of pooled sgRNA library using chemical transformation, electroporation is recommended (see “troubleshooting”).

2. Streak LB plates containing ampicillin with serial dilution of the transformed competent cells.
  a. Streak 10 μL of the 1 mL culture on the first plate.
  b. Dilute another 10 μL of the same culture in 90 μL of LB medium. Streak 10 μL of the first dilution on the second plate.
  c. Repeat step b to produce 10^2^, 10^3^, 10^4^, and 10^5^-fold dilutions. Grow the rest of the culture in 300 mL LB medium containing ampicillin 16—18 hr at 32 °C. Negative control is digested-vector-only-transformed cells.

***Note:*** The number of sgRNA library transformant colonies should be at least 10 times more than those of the negative control and at least 50 times the size of the library. The typical range of the number of library colonies obtained is 50,000 — 100,000. Extract plasmids using Macherey-Nagel Maxiprep kit.

**CRITICAL:** Do not proceed to the next step unless these criteria are met. See “troubleshooting.”

**Figure 1:**
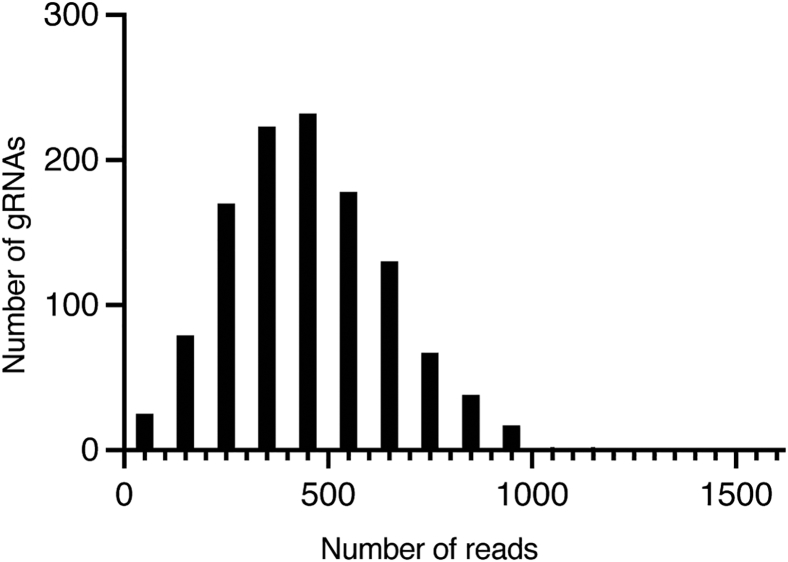
An example distribution of sequenced cloned library showing the number of sgRNAs and the number of sequencing reads

3. Sequence pooled sgRNA library on an Illumina sequencer. Confirm that the distribution of sgRNA library is symmetrically bell-shaped (Figure 1). For a library size of 1,000 sgRNAs, we performed 33 cycles of PCR and sequenced with a depth of 100,000 total reads.

### Ecotropic retrovirus preparation using Platinum-E packaging cell line

**Timing: 2 days**

It is important for an effective CRISPR screening to ensure that your virus titer allows decent transduction efficiency. Performing an optimization experiment using GeneJuice Transfection Reagent is recommended to account for user-to-user variables. On the other hand, ensuring delivery of a single vector copy per cell is critical. The target MOI is 0.25 (∼20% infected population), where no more than 3% of the cell population is infected with two or more viral particles. For a full-scale library transduction, the user should calculate the required number of transduced cells for optimal library representation (500 – 1,000× coverage^6^). For example, to establish 1,000× coverage for 1,000 sgRNA library, 10^6^ cells need to be transduced. With 20% transduction efficiency, a total of 5 × 10^6^ cells should be used. Additionally, to account for the engrafting efficiency in the recipient animals, we recommend the user to use at least four times the number of cells calculated above.

4. At day −1, seed 0.3 × 10^6^ Plat-E cells per well in a 6-well plate in 3 mL cell culture medium without antibiotics.
5. At day 0, follow the manufacturer’s instruction for GeneJuice Transfection Reagent. Use 2 μg pooled sgRNA library and 3 μL transfection reagent in 100 μL Opti-MEM medium per well.

**Figure 2:**
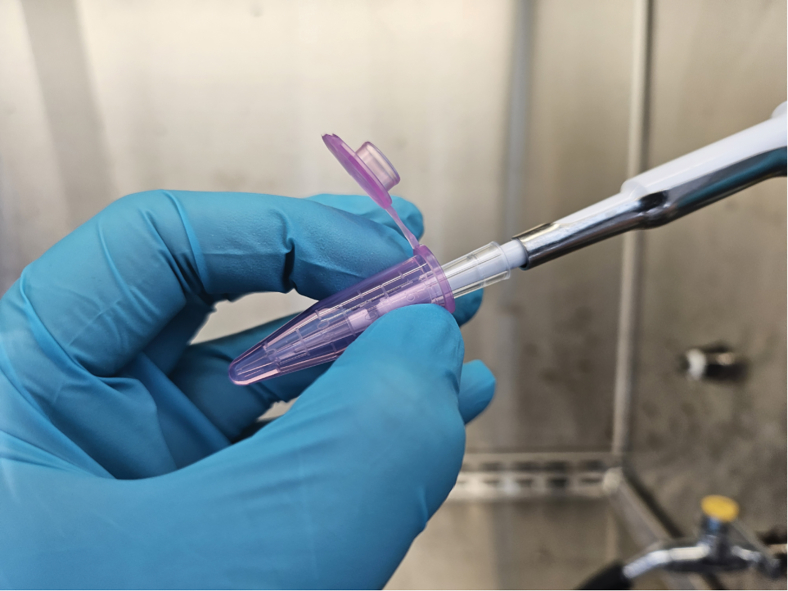
An illustration of dispensing the transfection reagent and the plasmids in the medium

**CRITICAL:** Slowly dispense the DNA and the reagent while keeping the pipette tip at least 2 mm below the surface of the liquid inside the Eppendorf tube and changing the tip’s positions every second (Figure 2).

6. At day 2, collect supernatant and freeze at −80 °C.

**Figure 3:**
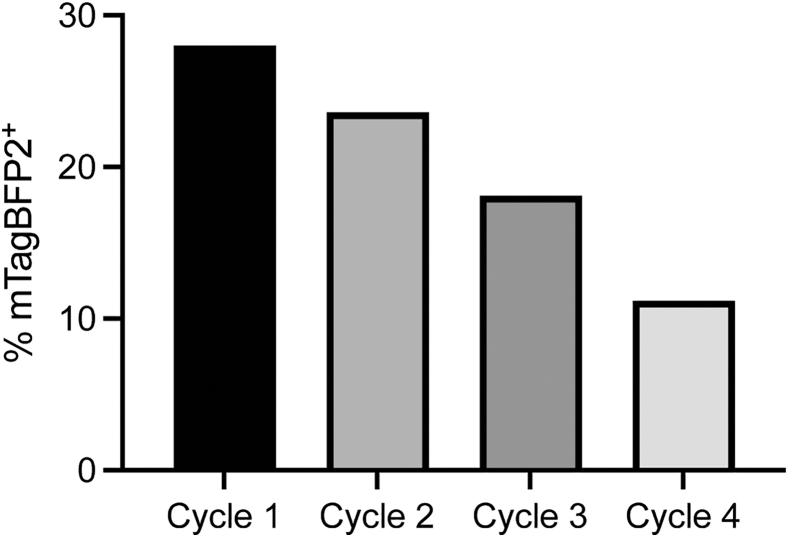
The effects of freeze-thaw cycles on transduction efficiency

***Note:*** Freeze-thaw cycles do not significantly decrease the transduction efficiency at least up to 3 cycles (Figure 3). Viral concentration step is not necessary. This protocol does not require usage of polybrene.

***Optional:*** Filter the supernatant through 0.45 μm polyethersulfone filter before freezing.

### Determination of retroviral titer

**Timing: 3 days**

Performing a small-scale viral titration experiment using the library pool virus prior to full-scale library infection is recommended. The goal is to determine the virus titer required to achieve a transduction efficiency within the range of 10 – 20% with primary mouse splenic B cells.

7. Plate and transduce activated mouse splenic B cells with different concentrations of retrovirus following step-by-step method details “transduction with pooled sgRNA library retrovirus.” Typically, we use viral media concentrations that are 50 — 100% of total media.
8. For analyzing the transduction efficiency by flow cytometry, note that the vector uses a mTag-BFP2 (Violet laser, 405 nm excitation wavelength).

### Experimental design

Performing a CRISPR screening experiment on GC B cells has been hindered by the lack of proper *in vitro* model that mimics GC B cells *in vivo*. Transducing sgRNA library into primary mouse B cells, which are then adoptively transferred and induced to express Cas9 upon recruitment into GCs in the recipient animals, overcomes the problem of testing the target genes in *de facto* GC B cells. As with other *in vivo* genetic screens such as those in tumor models, sufficient sgRNA recovery is critical for this protocol, which determines the size of an experiment, depending on the size of the library, the coverage, and the engraftment/recruitment efficiency. This protocol can also be utilized to test a single or a few target genes in a relatively short period of time as compared to alternative methods of creating new mouse strains, mouse breeding, or hematopoietic stem cell transduction followed by bone marrow transplant.

### Mouse strains and immunization

The following donor mouse model is designed to increase the experiment efficiency and reduce the cost. To delete target genes in antigen-specific B cells, Cre-inducible LSL-Cas9 mice^7^ were crossed to B1-8^hi^ mice^8^ (B1-8^hi^, LSL-Cas9). To restrict gene deletion to GC B cells, B1-8^hi^, LSL-Cas9 mice were crossed to mice that carry Cre transgene under the control of the promoter that expresses activation-induced cytidine deaminase (AID)^9^ (B1-8^hi^, LSL-Cas9, AID-Cre). In these mice, because they carry P2A self-cleaving peptide to co-express Cas9 and green fluorescent protein (GFP),^7^ deletion of loxP-flanked stop cassette induces the expression of the two proteins in antigen-experienced B cells. To enrich for antigen-specific B cells, B1-8^hi^, LSL-Cas9, AID-Cre mice were bred to mice that lack immunoglobulin kappa J region genes (B1-8^hi^, LSL-Cas9, AID-Cre, Kappa KO). The usage of these mice reduces the required amount of the retrovirus for transduction.

**Figure 4:**
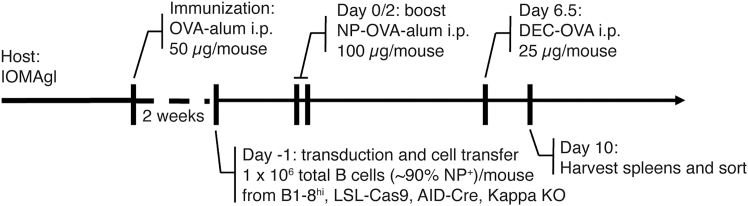
Immunization experiment scheme for in vivo screen Host mice (IOMA gl) were immunized with OVA in alum. At week 2 after immunization, B cells from B1-8^hi^, LSL-Cas9, AID-Cre, Kappa KO animals were transduced with pooled sgRNA library retrovirus and adoptively transferred to the hosts. Subsequently, the host animals were boosted with NP-OVA in alum at day 0 and 2 and administered with anti-DEC205-OVA mAb at day 6.5. Spleens were harvested and processed for cell sorting at day 10.

The choice of recipient mouse strain is critical for this protocol as it significantly influences the size of output sample and experiment. Adoptively transferring the transduced B cells into mice that contain a knocked-in germLine antibody IOMA gl, which is a precursor to a broadly-neutralizing HIV antibody of IOMA-class, allows enhanced recruitment of the transferred B cells into GCs after immunization with 4-hydroxy-3-nitro-phenylacetyl (NP) hapten-conjugated ovalbumin (NP-OVA) (Figure 4), because of the reduced competition with the endogenous B cell population for recruitment. Based on the number of transferred cells per recipient animal (∼1 × 10^6^ cells) and the total number of cells required for screening (see “before you begin”: “ecotropic retrovirus preparation using Platinum-E (Plat-E) packaging cell line”), calculate the number of animals for each experimental condition needed for the screen.

## Key resources table

**Table:** 

REAGENT or RESOURCE	SOURCE	IDENTIFIER
**Antibodies**		

Biotin-conjugated anti-mouse CD38 mAb (clone 90) (1 μg/mL)	eBioscience	cat# 13-0381-82
Biotin-conjugated anti-mouse IgD mAb (clone 11-26c (11-26)) (1 μg/mL)	eBioscience	cat# 13-5993-85
BV480-conjugated anti-mouse CD45R/B220 (clone RA3 6B2) (1 μg/mL)	BD	cat# 565631
Alexa 700-conjugated anti-mouse CD38 mAb (clone 90) (1 μg/mL)	eBioscience	cat# 56-0381-82
BV605-conjugated anti-mouse CD95 mAb (clone SA367H8) (1 μg/mL)	Biolegend	cat# 152612
Anti-DEC205-OVA chimeric mAb	Boscardin et. al.^10^	N/A
Anti-mouse CD16/CD32 mAb (clone 2.4G2) (1 μg/mL)	BD	cat# 553142

**Bacterial and virus strains**		

NEB Stable Competent E. coli, High Efficiency	New England Biolabs	cat# C3040H

**Chemicals, peptides, and recombinant proteins**		

Zombie NIR Fixable Viability Kit	Biolegend	cat# 423105
GeneJuice Transfection Reagent	Millipore Sigma	cat# 70967
NEBuilder HiFi DNA Assembly Master Mix	New England Biolabs	cat# E2621
BbsI-HF	New England Biolabs	cat# R3539
MegaCD40L™ (soluble) (mouse)	Enzo Life Sciences	cat# ALX-522-120-C010
CpG ODN2395	Invivogen	cat# tlrl-2395
Human BAFF (BLyS) Recombinant Protein	PeproTech	cat# 310-13
Alhydrogel	Invivogen	cat# vac-alu
Pellet Paint NF Co-Precipitant	Millipore Sigma	cat# 70748
Phusion Green HSII HF DNA Polymerase	Thermo Fisher Scientific	cat# F537
OVA	Biosearch Technologies	cat# O-1000
NP-OVA (conjugation ratio: 21)	Biosearch Technologies	cat# N-5051
Opti-ME I Reduced Serum Medium, GlutaMAX Supplement	Gibco	cat# 51985034

**Critical commercial assays**		

Anti-CD43 microbeads	Miltenyi Biotec	cat# 130-049-801
Anti-Ter-119 microbeads	Miltenyi Biotec	cat# 130-049-901
Strepavidin microbeads	Miltenyi Biotec	cat# 130-048-101
QIAamp DNA Blood Mini Kit	Qiagen	cat# 51104
LS columns	Miltenyi Biotec	cat# 130-042-401
QuadroMACS Separator	Miltenyi Biotec	cat# 130-090-976
MACS MultiStand	Miltenyi Biotec	cat# 130-042-303

**Experimental models: Cell lines**		

Platinum-E Retroviral Packaging Cell Line, Ecotropic	Cell Biolabs	cat# RV-101

**Experimental models: Organisms/strains**		

Mouse: 6-weeks-old, sex-matched LSL-Cas9: B6J.129(B6N)-Gt(ROSA)26Sortm1(CAG-cas9∗,-EGFP)Fezh/J	The Jackson Laboratory	cat# 026175
Mouse: 6-weeks-old, sex-matched B1-8hi: B6.129P2-Ptprca Ightm1Mnz/J	The Jackson Laboratory	cat# 007594
Mouse: 6-weeks-old, sex-matched AID-Cre: B6.129P2-Aicdatm1(cre)Mnz/J	The Jackson Laboratory	cat# 007770
Mouse: 6-weeks-old, sex-matched Kappa KO	This paper	N/A
Mouse: 6-weeks-old, sex-matched IOMA gl	Gristick et. al.^11^	N/A

**Oligonucleotides**		

Primer: Sequencing primer: TTTTCAAGTTGATAACGG ACTAGCCTTATTTAAACTTGCTATGCTGTTTC CAGCATAGCTCTTAAAC	Zhu et. al.^2^	N/A
Primer: Index primer: TTTCAAGTTACGGTAAGCATAT GATAGTCCATTTTAAAACATAATTTTAAAACTG CAAACTACCCAAGAAA	Zhu et. al.^2^	N/A
Primer: Amplification primer forward with index 1 (TAAGGC): CAAGCAGAAGACGGCATACGAGATCTAAGGCTTTC TTGGGTAGTTTGCAGTTTT	This paper	N/A
Primer: Amplification primer forward with index 2 (CGTACT): CAAGCAGAAGACGGCATACGAGATCCGTACTTTTC TTGGGTAGTTTGCAGTTTT	This paper	N/A
Primer: Amplification primer forward with index 3 (AAGAGG): CAAGCAGAAGACGGCATACGAGATCAAGAGGTTTC TTGGGTAGTTTGCAGTTTT	This paper	N/A
Primer: Amplification primer reverse: AATGATACGGCGA CCACCGAGATCTACACCCTTCTGCTACGTCCCTTCG	This paper	N/A
Plasmid: pMP71-sgEmpty	This paper	N/A

**Software and algorithms**		

Prism 10	GraphPad	https://www.graphpad.com
FlowJo v.10	FlowJo, LLC	https://www.flowjo.com
MAGeCK	Li et. al.^3^	https://pubmed.ncbi.nlm.nih.gov/25476604/
Geneious Prime v.2025	Geneious	https://www.geneious.com

**Other**		

NucleoBond Xtra Maxi	Macherey-Nagel	cat# 740414.100
NucleoSpin Gel and PCR Clean-up	Macherey-Nagel	cat# 740609
BD FACS Aria II	BD	N/A

## Step-by-step method details

### OVA-prime the recipient mice

**Timing: 2 weeks**

**CRITICAL:** Follow governmental and institutional guidelines and regulations. Perform experiments in compliance with protocols approved by local animal ethics committees.

Immunize the recipient mice with OVA two weeks in advance of adoptively transferring the library-transduced B cells. This step ensures consistent recruitment of the transferred cells into GCs upon boosting with NP-OVA later.

1. Prepare 50 μg OVA per animal in 66 μL PBS and mix with 34 μL Alhydrogel while rotating (10 rpm) at 4 °C for 30 min. Inject by an intraperitoneal (i.p.) route 100 μL OVA/Alhydrogel into the recipient mice.

***Optional:*** Confirm successful immunization by measuring anti-OVA serum titer at week 2 after immunization before proceeding to the next step.

### Transduction with pooled sgRNA library retrovirus

**Timing: 4 days**

Deliver pooled sgRNA library into activated primary mouse B cells. Mitogenic activation of these cells creates the optimal condition for spinfection.

2. At day 0, euthanize mice according to institutional guidelines.
3. Harvest spleens from donor mice.
  a. Mince the spleens and pass through a 70 μm cell strainer to generate single cell splenocyte suspensions.
  b. Count the cells.

***Note:*** Usage of enzymes is not necessary.

4. Spin down the cells at 300 × *g* at 4 °C for 5 min. Resuspend 1 × 10^8^ cells/800 μL FACS buffer (2% fetal bovine serum (FBS) and 2mM EDTA in PBS).
5. Add 100 μL CD43 microbeads and 100 μL Ter-119 microbeads per 800 μL cell suspension and incubate at 4 °C for 15 min while rotating the tubes.
6. Wash the cells with 10 mL FACS buffer. Spin down at 300 × *g* at 4 °C for 5 min. Resuspend the cells in 3 mL FACS buffer.
7. Isolate B cells following the manufacturer’s instructions for Miltenyi microbeads.
  a. Apply the cell suspension onto LS columns in QuadroMACS Separator.
  b. Collect unlabeled cells that pass through.
  c. Wash column with 3 mL FACS buffer twice while collecting total effluent.
  d. Count and spin down the cells at 300 × *g* at 4 °C for 5 min.
8. Plate 25 × 10^6^ splenic B cells in 10-cm dish in 10 mL B-cell culture medium (10% FBS, non-essential amino acids, HEPES, anti-biotic/anti-mycotic, 2-mercaptoethanol in RPMI) containing 100 ng/mL MegaCD40L protein, 5 μg/mL CpG, and 40 ng/mL human BAFF.
9. At day 2, wash activated B cells in 10 mL PBS and count.

**Figure 5:**
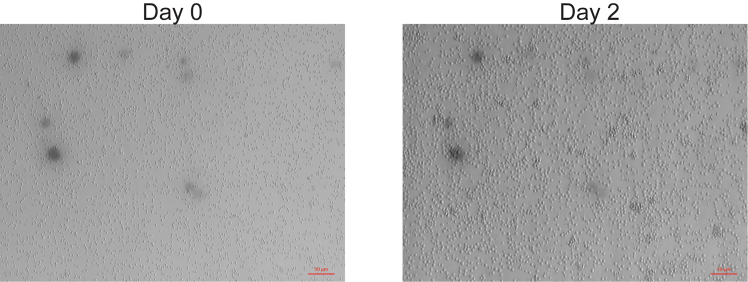
Photographs of B cells before (day 0) and after activation (day 2) Scale bars, 50 μm.

**CRITICAL:** The cells should become approximately 1.3–1.5-fold larger in diameter than day 0 (Figure 5). The extent of cell growth determines the efficiency of spinofection.

10. Plate 5 × 10^6^ activated B cells per well in a 6-well plate in 2 mL Plat-E cell culture medium. Spin the plate at 1,500 × *g* at 30 °C for 10 min.

**CRITICAL:** The temperature during spinofection affects the transduction efficiency. Do not spin at 4 °C. If necessary, pre-warm the centrifuge before step 9. See “troubleshooting.”

***Note:*** Transduce enough cells to collect an input sample on the day of adoptive transfer. For 1,000 sgRNA library and 20% transduction efficiency, seed for an input at least 5 × 10^6^ cells to be able to sort 1 × 10^5^ transduced cells on the day of adoptive transfer (100× coverage), accounting for some loss of cells throughout the procedure.

**Figure 6:**
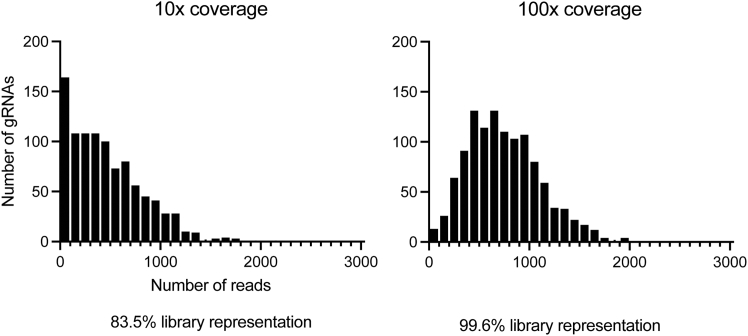
Pooled sgRNA library representation at 10x and 100x coverage

***Note:*** Although the highest standard is 500—1,000× coverage of the library, 100—500× coverage will still be effective,^6^^,^^12^ which becomes important when cell number are limited. We observed that 100× coverage does not lead to a significant loss of library diversity (Figure 6).

11. Carefully aspirate supernatant. Add 2 mL retrovirus per well and spin the plate at 1,500 × *g* at 30 °C for 45 min.

**CRITICAL:** Add the first 1 mL virus by slowly dispensing it down the inner wall of the well at 5 mm above the bottom surface. Then, slowly dispense the next 1 mL while keeping the pipette tip at least 1—2 mm below the surface of the liquid. Check to ensure that most of the cells are still attached to the bottom of the well after adding the virus.

12. Add 2 mL B-cell culture medium containing 200 ng/mL MegaCD40L protein, 10 μg/mL CpG, 80 ng/mL human BAFF, and 110 μM 2-mercaptoethanol.
13. At day 4, wash transduced B cells in 10 mL FACS buffer and analyze the cells for the expression of mTagBFP2 by flow cytometry (Violet laser, 405 nm excitation wavelength).
14. Once the infection rate has been confirmed (see “before you begin”: “determination of retroviral titer”), wash the cells in 10 mL PBS for adoptive transfer.

### Immunization and *in vivo* screen

**Timing: 11 days**

Adoptively transfer the pooled sgRNA library-transduced B cells into recipient mice and immunize them with NP-OVA for the screen. Our group typically injects intravenous (i.v.) ∼1 × 10^6^ cells in PBS per animal.

15. Resuspend the transduced cells in 10 mL PBS and count. Set aside the cells for an input sample.

***Note:*** Keep cells on ice to maintain cell viability.

16. Adjust the cell suspension volume to be 1 × 10^6^ cells/100 μL. Inject i.v. 100 μL cell suspension into each animal at day −1.
17. Sort input sample for mTagBFP2^+^ cells. Spin down the sorted cells at 300 × *g* at 4 °C for 5 min.

***Note:*** The genomic DNA can be extracted immediately (see steps 35 – 42 below), or the cell pellet can be kept frozen at −80 °C for the duration of the experiment.

18. At day 0, immunize the recipient mice with NP-OVA.
  a. Prepare 100 μg NP-OVA per animal in 66 μL PBS and mix with 34 μL Alhydrogel while rotating (10 rpm) at 4 °C for 30 min.
  b. Inject i.p. 100 μL NP-OVA/Alhydrogel into the recipient mice.
19. Repeat step 18 at day 2.
20. At day 6.5 (84 h before harvest), inject i.p. 25 μg anti-DEC205-OVA chimeric monoclonal antibody (mAb) in 100 μl PBS into the recipient mice.

**CRITICAL:** Anti-DEC205-OVA mAb targets a cell-surface lectin DEC205 and is fused to OVA protein. Its injection delivers antigens to GC B cells, which results in their expansion.^13^ It is important to wait 84 h after the injection to ensure a normal light zone to dark zone GC B cell ratio as the mAb initially alters their migration pattern.^13^

### Spleen harvesting and genomic DNA extraction from an *in vivo* screen

**Timing: 1 day**

Process the spleens and sort for the pooled sgRNA library-transduced GC B cells before gDNA extraction. B cell isolation protocol is modified from steps 5—7 in step-by-step method details “transduction with pooled sgRNA library retrovirus” to enrich for GC B cells to shorten the sorting time.

**Figure 7:**
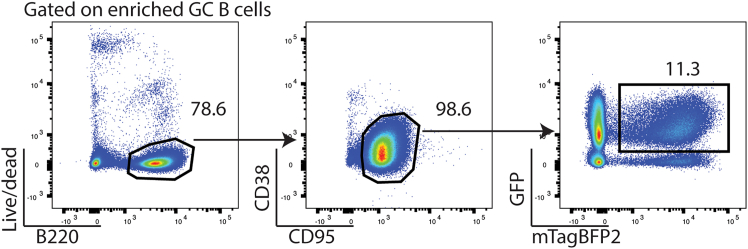
Gating strategy for sorting transduced GC B cells Among live/dead^-^, B220^+^ B cells, GC B cells were gated as CD38^-^, CD95^+^ cells. GFP^+^, mTagBFP2^+^ GC B cells were sorted.

21. At Day 10 after NP-OVA immunization, euthanize mice according to institutional guidelines.
22. Harvest the spleens from the recipient mice.
  a. Mince the spleens and pass through a 70 μm cell strainer to generate single cell splenocyte suspensions.
    ***Note:*** Usage of enzymes is not necessary.
  b. Count the cells.
    ***Note:*** The typical range of the number of cells is 0.3–1 × 10^8^ cells per spleen.
23. Spin down the cells at 300 × *g* at 4 °C for 5 min. Resuspend 1 × 10^8^ cells/1 mL FACS buffer.
24. Add 1 μg anti-IgD mAb and 1 μg anti-CD38 mAb per 1 mL cell suspension. Incubate at 4 °C for 15 min while rotating the tubes.
25. Wash the cells with 10 mL FACS buffer. Spin down at 300 × *g* at 4 °C for 5 min.
26. Resuspend 1 × 10^8^ cells/700 μL FACS buffer.
27. Add 100 μL each of CD43, Ter-119, and Streptavidin microbeads per 700 μL cell suspension. Incubate at 4 °C for 15 min while rotating the tubes.
28. Wash the cells with 10 mL FACS buffer. Spin down at 300 × *g* at 4 °C for 5 min.
29. Isolate GC B cells following the manufacturer’s instructions for Miltenyi microbeads.
30. Spin down the cells at 300 × *g* at 4 °C for 10 min.
  a. Resuspend 1 × 10^8^ cells/200 μL FACS buffer containing Fc-blocking antibody (anti-CD16/CD32 mAb) at 1 μg/mL.
  b. Incubate at 4 °C for 15 min.
31. Prepare antibody staining mixture containing Zombie NIR live/dead dye at 1:250 dilution and anti-B220, anti-CD38, and anti-CD95 mAb all at 2 μg/mL.
  a. Add 200 μL staining mixture/200 μL cell suspension.
  b. Incubate at 4 °C for 15 min.
32. Wash the cells with 10 mL FACS buffer.
33. Sort for mTagBFP2^+^ GC B cells (live/dead^-^, B220^+^, CD38^-^, CD95^+^) at 10,000 events/sec using 70 μm nozzle (BD FACS Aria II) in 1.5-mL Eppendorf tubes containing 300 l PBS (Figure 7). Spin down at 300 × *g* at 4 °C for 5 min.

**CRITICAL:** For 1,000 sgRNA library, the user is recommended to collect at least 5 × 10^5^ cells per replicate for the total of at least 3—4 replicates. Each replicate is pooled from 20 mice.

**Pause point:** After spinning down and removing the supernatant, the cell pellets can be stored at −80 °C until the user is ready to extract gDNA.

34. Remove the frozen input and output sample pellets from the −80 °C freezer and thaw at 20—25 °C.
35. Extract gDNA from cell pellets using QIAamp DNA Blood Mini Kits following the manufacturer’s instructions.
  a. Add 4 μL RNase A stock solution (100 mg/mL)/200 μL PBS with 10^6^ cells before starting the gDNA extraction.
  b. Load up to 10^6^ cells per column.
  c. During the elution step, incubate the columns loaded with 50 μL nuclease-free water at 20—25 °C for 5 min before centrifugation. Repeat elution once more and combine with the first elution.

***Note:*** It is recommended to concentrate the eluate by ethanol precipitation before performing PCR amplification.

36. Add 2 μL Pellet Paint to the eluate followed by 0.1 volume of 3 M sodium acetate. Mix by vortexing.
37. Add 2 volume of ethanol and vortex. Incubate at 20—25 °C for 2 min.
38. Spin down at 16,000 × *g* at 4 °C for 5 min. Carefully remove supernatant.
39. Wash with 70% ethanol and vortex.
40. Repeat step 38. Wash with 100% ethanol and vortex.
41. Repeat step 38. Air-dry residual ethanol and dissolve the pellet with 12 μL nuclease-free water for up to 10^6^ sorted cells.
42. Measure the concentration of gDNA using Nanodrop or Qubit machine.

**CRITICAL:** The total yield of gDNA should be at least 100 – 500 ng per 10^5^ cells. See “troubleshooting.”

**Pause point:** gDNA can be stored at 4 or −80 °C.

### Sequencing and data analysis from an *in vivo* screen

**Timing: 2–3 days**

PCR amplify sgRNA sequences from gDNA for high-throughput sequencing and analysis.

43. Prepare PCR master mix.

**Table:** 

Reagent	Final concentration	Amount
5× Phusion Green HF buffer	1×	4 μL
dNTP (1 mM)	200 μM	4 μL
Forward primer (5 μM)	500 nM	2 μL
Reverse primer (5 μM)	500 nM	2 μL
DMSO	–	0.6 μL
gDNA	–	7.2 μL
Phusion Hot Start II (2 U/μL)	0.02 U/μL	0.2 μL

**CRITICAL:** For 1,000 sgRNA library, the user is recommended to use at least 500 ng for downstream PCR amplification.

**Table:** 

Steps	Temperature	Time	Cycles
Initial Denaturation	98°C	1 min	1
Denaturation	98°C	10 s	33 cycles
Annealing	62.5°C	30 s	–
Extension	72°C	15 s	–
Final extension	72°C	5 min	1
Hold	4°C	forever	forever

44. Run PCR reaction mixture on 2% agarose gel with DNA ladder.
45. Excise the band at 500 bp for DNA purification by gel extraction, following the manufacturer’s instructions. Non-specific bands are not typically observed.
46. Measure the concentration of PCR product using Nanodrop or Qubit machine. The expected amount of PCR product is approximately 300—1,000 ng.
47. Sequence the libraries on an Illumina sequencer. For a library size of 1,000 sgRNAs, we performed 33 cycles of PCR and sequenced with a depth of 400 million total reads.
48. Use Geneious or other equivalent programs to map the reads to the reference sgRNA library sequences. Create counts table by exporting documents.
49. Use MAGeCK to calculate statistical enrichment and depletion of the genes in the screen by providing the counts table as input.

***Optional:*** Perform normalization using --control-sgrna option.

## Expected outcomes

We use this protocol to generate populations of GC B cells that have deleted genes targeted by the CRISPR-Cas9 system. It is expected that these genes should be deleted in the activated B cells prior to their differentiation into GC B cells when the AID gene promoter becomes active. This allows for *in vivo* pooled CRISPR screens of genes critical for GC B cell differentiation, dynamics, and functions as well as oncogenes and tumor suppressors in the context of GC-derived lymphomas. Moreover, this protocol can be relatively easily modified to study other B cell populations such as plasma cells by using Blimp-Cre or Blimp-CreERT2 model, instead of AID-Cre.

**Figure 8:**
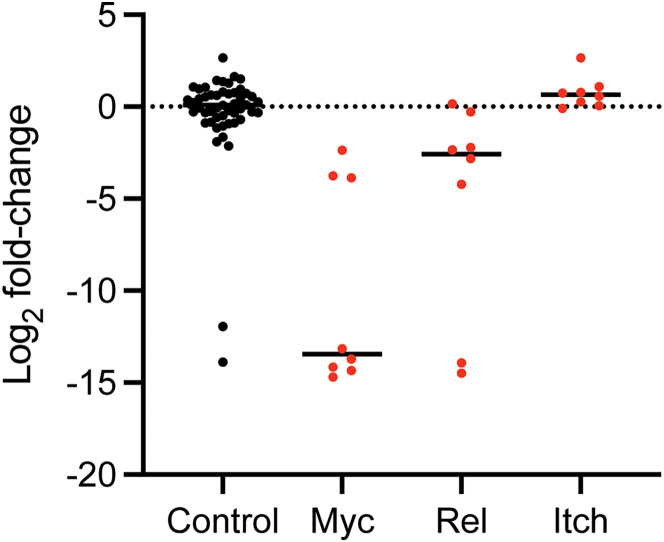
An example of screening results The log_2_ fold changes of sgRNA counts are plotted for control (black) and hit genes (red). Each dot represents a sgRNA. Bars indicate median values.

**Figure 9:**
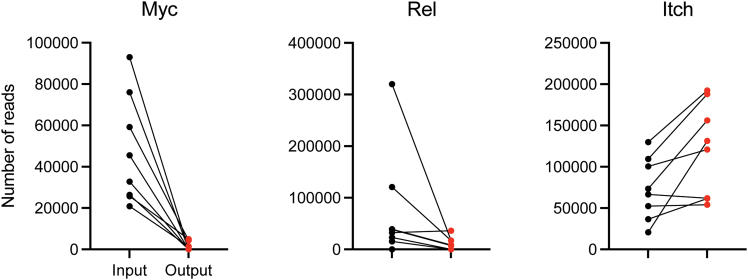
The number of detected sgRNAs targeting each hit gene

In a screen testing a subset of metabolic genes, we identified Myc and Rel as two of the top ranked genes for which the targeting sgRNAs were significantly depleted in GC B cells (FDR < 0.01 for 8 and 4 out of 8 sgRNAs, respectively) (Figure 8). This is consistent with previous studies showing their critical roles in these cells.^14^^,^^15^^,^^16^ Moreover, similar to the findings that B cells deficient in Itch gene constituted disproportionately larger fraction of GCs compared to wild-type B cells,^17^ sgRNAs targeting Itch were significantly enriched in GC B cells (FDR < 0.05 for 3 out of 8 sgRNAs) (Figure 8). The number of detected sgRNAs targeting each gene is shown in Figure 9.

## Limitations

This protocol was designed to delete genes prior to the onset of GC B cell differentiation, and therefore it is not ideal for studying GC maintenance or exit. However, performing the screens at multiple time points and comparing among various B cell subsets may provide initial hypothesis to be further tested using other genetic models. Additionally, the outcome of the screens will be limited to genes with cell-intrinsic effects.

## Troubleshooting

### Problem 1

Incomplete representation of pooled sgRNA library after cloning and sequencing the plasmids (from “pooled sgRNA library cloning”).

### Potential solution

• Ensure that the read depth is at least 100 times the size of the library.
• Optimize PCR conditions so that the minimum number of PCR cycle is used.
• Transform electrocompetent bacteria.

### Problem 2

No mTagBFP2 expression is observed after transduction with pooled sgRNA library retrovirus (from “transduction with pooled sgRNA library retrovirus”).

### Potential solution

• Optimize Plat-E cell density and ratio of GeneJuice transfection reagent to DNA for transfection.
• Make sure to pre-warm the centrifuge to 30 °C for transduction.
• After spinning the cells at 1,500 *g* for 10 min before spinofection, confirm that most cells are attached to the bottom of the plate.

### Problem 3

The proportion of mTagBFP2+ GC B cells is significantly lower than that initially observed after transduction (from “spleen harvesting and gDNA extraction from an in vivo screen”).

### Potential solution

• Re-make library cells and repeat adoptive transfer/immunization.
• Backcross the recipient mice to the background of the donor animals and confirm genotype by SNP testing.
• Ensure that the recipient mice are OVA-primed. Confirm anti-OVA serum titer before the adoptive transfer of the transduced cells.

### Problem 4

Low gDNA yield (from “spleen harvesting and gDNA extraction from an in vivo screen”).

### Potential solution

• Move quickly from spleen harvest to sorting to reduce cell death and stress. The expected viability is approximately 80—90%.
• Incubate Qiagen columns with warmed nuclease-free water for up to 30 min before spinning with caps closed.
• During ethanol precipitation and washing steps, ensure that DNA pellet is intact while removing supernatant.

### Problem 5

Inability detect band after gDNA PCR (from “sequencing and data analysis from an in vivo screen”).

### Potential solution

• After ethanol precipitation and washing, ensure the pellet is completely dried before dissolving it in water.
• Confirm the amount of gDNA used for PCR reaction (e.g., 500 ng for 1,000 sgRNA library).
• Increase the number of PCR cycles.

## Resource availability

### Lead contact

Further information and requests for resources and reagents should be directed to and will be fulfilled by the lead contact, Jun Hong (jhong@rockefeller.edu).

### Technical contact

Technical questions on executing this protocol should be directed to and will be fulfilled by the lead contact, Jun Hong (jhong@rockefeller.edu).

### Materials availability

Plasmids generated in this study are available on request. The mouse lines obtained from other laboratories are described in the key resources table and may require a material transfer agreement (MTA) with the providing scientists.

### Data and code availability

Additional data described in this manuscript are available from the lead contact, Jun Hong (jhong@rockefeller.edu) upon reasonable request.

## Acknowledgments

We thank T. Eisenreich for help with mouse colony management, K. Yao for technical help, The Rockefeller University Flow Cytometry Resource Center for cell sorting, The Rockefeller University Transgenic and Reproductive Technology Center for mouse colony expansion and accelerated breeding, all the members of the Nussenzweig laboratory for discussion, Dr. H. Hartweger for IOMA gl mice, Dr. A. Escolano for Kappa KO mice, Dr. J. Merkenschlager for the pMP71 plasmid, Dr. A. Gazumyan for anti-DEC205-OVA chimeric mAb production, and Dr. G. Unlu for the assistance with the library design. This work was supported by 10.13039/100000002NIH (2P01AI100148), NIH (UM1AI144462), NIH (UM1AI191237), and the Stavros Niarchos Foundation Institute for Global Infectious Disease Research to M.C.N. M.C.N. is an 10.13039/100000011HHMI investigator. The graphical abstract was created using Biorender.com.

## Author contributions

J.P.H. and M.C.N. contributed to the study design. J.P.H. performed experiments and wrote the manuscript. M.C.N. supervised the work.

## Declaration of interests

The authors declare no competing interests.
